# Gene Duplication Accelerates the Pace of Protein Gain and Loss from Plant Organelles

**DOI:** 10.1093/molbev/msz275

**Published:** 2019-11-21

**Authors:** Rona Costello, David M Emms, Steven Kelly

**Affiliations:** Department of Plant Sciences, University of Oxford, Oxford, United Kingdom

**Keywords:** evolution, organelle, plant, proteome, protein, targeting, duplication, localization

## Abstract

Organelle biogenesis and function is dependent on the concerted action of both organellar-encoded (if present) and nuclear-encoded proteins. Differences between homologous organelles across the Plant Kingdom arise, in part, as a result of differences in the cohort of nuclear-encoded proteins that are targeted to them. However, neither the rate at which differences in protein targeting accumulate nor the evolutionary consequences of these changes are known. Using phylogenomic approaches coupled to ancestral state estimation, we show that the plant organellar proteome has diversified in proportion with molecular sequence evolution such that the proteomes of plant chloroplasts and mitochondria lose or gain on average 3.6 proteins per million years. We further demonstrate that changes in organellar protein targeting are associated with an increase in the rate of molecular sequence evolution and that such changes predominantly occur in genes with regulatory rather than metabolic functions. Finally, we show that gain and loss of protein target signals occurs at a higher rate following gene duplication, revealing that gene and genome duplication are a key facilitator of plant organelle evolution.

## Introduction

A hallmark of eukaryotic cells is the compartmentalization of intracellular processes into specialized membrane-bound compartments known as organelles. Plant cells contain several such organelles, including the nucleus, chloroplast, mitochondrion, peroxisome, Golgi, endoplasmic reticulum, and vacuole. With the exception of the chloroplast and mitochondrion, all organelle proteins are encoded in the nucleus of the cell and must be imported from the cytosol via import channels on the organellar membrane. For both the chloroplast and mitochondrion, a fraction of their respective proteomes are encoded by their own organellar genomes; however, the vast majority of chloroplast and mitochondrial proteins are encoded in the nucleus (Timmis et al. 2004).

Nuclear-encoded organellar proteins are translocated to and across the organellar membrane by means of a short, often cleavable, targeting sequence located within the amino acid sequence of the protein (Schatz and Dobberstein 1996). Although these target peptides come in a variety of forms, for proteins of the chloroplast, mitochondrion, and secretory organelles they are usually located at the N-terminus of the polypeptide chain and cleaved upon entry into the organelle (Kunze and Berger 2015). As such, these peptides, once removed, have no impact on the final function of the mature protein. In addition, there is substantial flexibility in the sequence and length of targeting peptides (Bannai et al. 2002) such that a large diversity of sequences can function to target proteins to their intended destination.

From early in the investigation of the protein content of organelles it was noted that many homologous proteins had divergent subcellular localizations, both within and between species, for example, the cytosolic and mitochondrial isoforms of phosphoenolpyruvate carboxykinase proteins in animals (Nordlie and Lardy 1963) or the cytosolic and chloroplastic isoforms of sugar phosphate enzymes in plants (Schnarrenberger et al. 1983). Following the advent of protein, cDNA, and genome sequence data, it was realized that disparate organellar localization of these proteins was facilitated by differences in the presence and absence of N-terminal target signals, and that such differences occur among many homologous proteins in different species (Marques et al. 2008; Qian and Zhang 2009; Wang et al. 2009; Liu et al. 2014; Ren et al. 2014). Furthermore, larger scale bioinformatic analysis of plant gene families has suggested that changes in protein targeting of homologous genes may be a common occurrence during plant evolution (Heilmann et al. 2004; Richly and Leister 2004). However, the extent to which organellar proteomes have diverged over time through such changes in targeting is unknown. Although many examples of disparate organellar targeting within gene families have been identified, it is unknown how these changes impact the global regulatory and metabolic landscape of organelles. Furthermore, the extent to which the occurrence of changes in organellar targeting is influenced by evolutionary events of the nuclear genome, such as gene and whole-genome duplication, remains an open question (Byun-McKay and Geeta 2007; Marques et al. 2008; McKay et al. 2009; Byun and Singh 2013).

To address these questions a phylogenomic approach, coupled with ancestral state estimation, was taken to interrogate the evolution of nuclear-encoded organellar proteomes across the land plant phylogeny. This uncovered a pattern of continual change, with ∼3.6 changes per million years to the nuclear-encoded proteomes of both the chloroplast and mitochondrion. Functional analysis of the genes encoding these proteins revealed that these changes occurred predominantly to genes with regulatory rather than metabolic functions, indicating that altered regulatory capacity is a major theme of organellar proteome evolution in plants. Changes in organellar targeting of proteins is also shown to be associated with an increase in the rate of molecular sequence evolution. Finally, this analysis demonstrated that changes in protein targeting occur at a higher rate following gene duplication, providing evidence that gene and genome duplication are key facilitators of plant organelle evolution.

## Results

### Widespread Gain and Loss of Organellar Targeting Signals Have Occurred throughout the Evolution of Plants

**FIg. 1.**
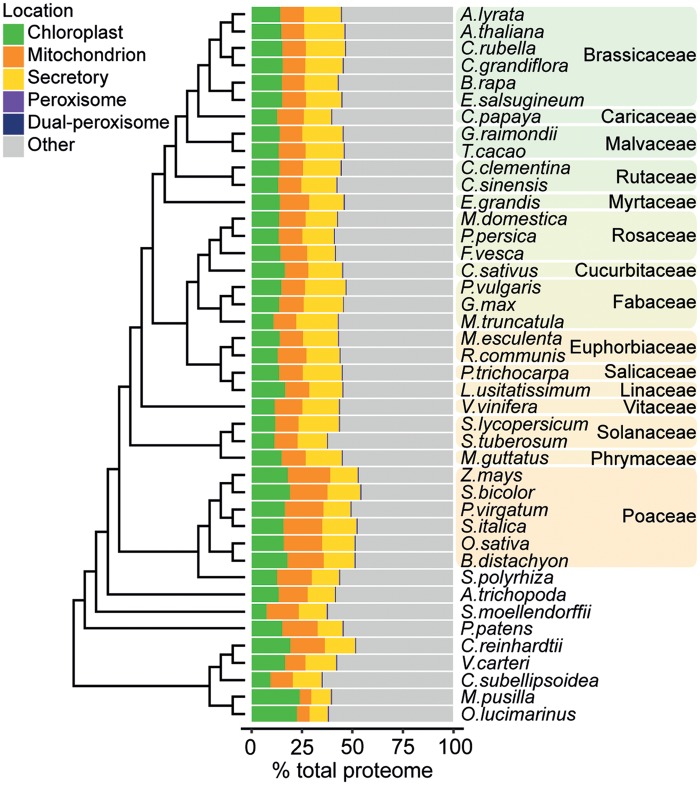
The predicted organelle proteome sizes for the 42 species in this data set as a percentage of total proteome size. Chloroplast, mitochondrion, and secretory pathway proteins were predicted using TargetP and PredAlgo. Peroxisomal proteins were identified by the presence of a peroxisomal targeting signal 1 or 2 (PTS1 or PTS2). Proteins predicted to be organellar by TargetP but which also contained a PTS1 or PTS2 were assigned as dual-localized peroxisomal proteins (*n* = 2,973).

A bioinformatic approach was taken to build organelle proteomes for the chloroplast, mitochondrion, secretory organelles, and peroxisome of 42 diverse plant species. On average across land plants, the predicted chloroplast, mitochondrion, secretory, and peroxisome proteomes comprised 14% (±2%), 14% (±3%), 17% (±2%), and 0.32% (±0.05%) of the total proteome, respectively (fig. 1 and supplementary file S2, Supplementary Material online). Here, the secretory proteome was considered to comprise all proteins with a signal peptide (SP). However, it should be noted that the secretory pathway itself is made up of multiple organelles including the endoplasmic reticulum and Golgi apparatus, and the final destination of proteins harboring an SP may be either of these two organelles, the cell membrane or secretion into the extracellular space.

**FIg. 2.**
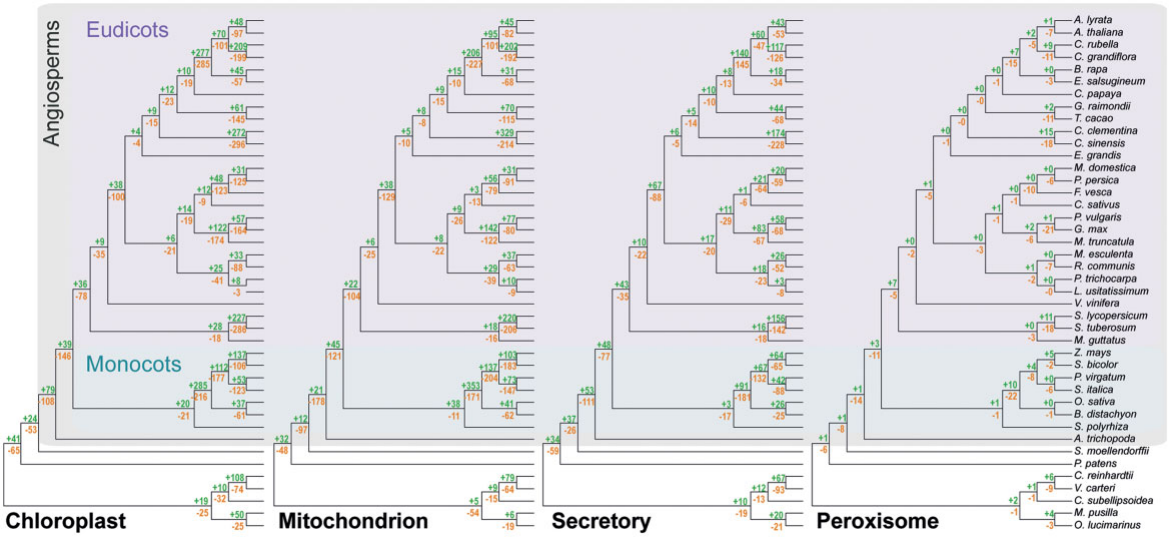
The number of gains (green) and losses (orange) in organellar protein targeting to the chloroplast, mitochondrion, secretory pathway, and peroxisome identified to have occurred along each nonterminal branch of the species tree encapsulated by the species used in this study. Branch lengths shown in the figure are not drawn to scale and do not correspond to evolutionary distances.

To identify the changes in nuclear-encoded protein targeting (and therefore organellar proteome content) that have occurred during the evolution of these species, the predicted localization of proteins was combined with the complete set of species-tree-reconciled gene trees (*n* = 18,823) for all orthogroups (gene families) of this data set. Ancestral state estimation was then performed to predict the subcellular localization of the ancestral proteins represented by each internal node of each reconciled gene tree. Evolutionary changes in protein localization were then identified and mapped to the corresponding branch of the species tree to infer the number of changes in protein targeting that occurred to each organelle along each branch of the species tree (see Materials and Methods). In total, across the four organelles, 6,162 gains and 9,058 losses were identified and mapped to internal branches of the species tree (fig. 2). Gains and losses in protein targeting were observed along every branch of the species tree, suggesting that changes in organellar localization have been a widespread phenomenon during plant evolution.

There are other mechanisms that can result in disparate localization of proteins that have not been considered in this study, for example, the roles of dual targeting and alternative gene splicing. Although alternative splicing is accepted as a wide-spread phenomenon in the plant genome, with more than 60% of intron-containing genes thought to undergo alternative splicing (Syed et al. 2012), there are only a handful of reports of it resulting in disparate localization of gene isoforms (de la Fuente van Bentem et al. 2003; Folli et al. 2010; An et al. 2017). Alternative protein localization for the same gene has also been reported for genes with alternative transcription start sites (Thatcher et al. 2007; Cabout et al. 2017). As alternative transcript variants were not considered in this study, it is likely that the findings presented here represent a conservative estimate of the extent to which changes in protein targeting occur.

### Changes to the Organellar Proteome Occurred Continuously throughout Plant Evolution

**FIg. 3.**
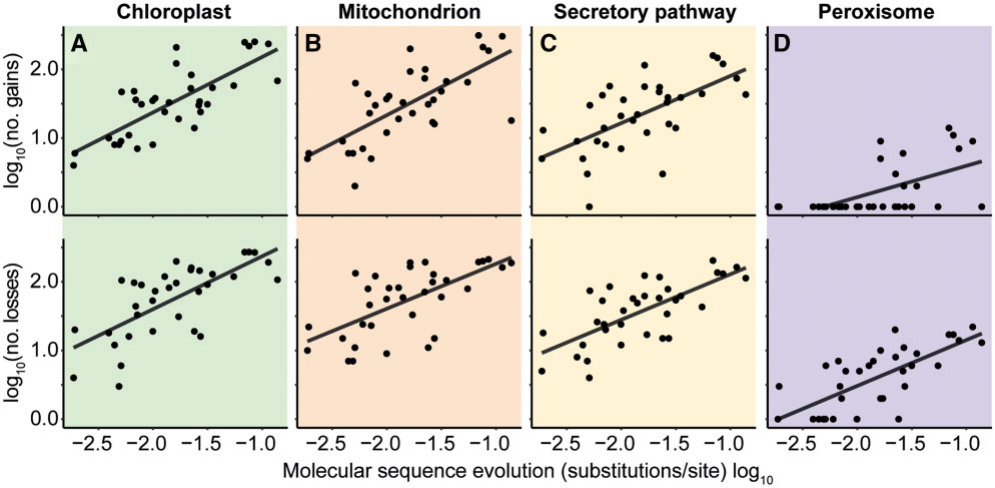
The relationship between molecular sequence evolution and organellar proteome evolution. There was a positive relationship between species-tree branch length (amino acid substitutions per site) and the number of gains or losses to (*A*) the chloroplast (*R*^2^ = 0.59, 0.49), (*B*) the mitochondrion (*R*^2^ = 0.50, 0.42), (*C*) the secretory pathway (*R*^2^ = 0.40, 0.50). All correlations *P* < 0.001. (*D*) Fewer gains and losses were observed in peroxisomal targeting, with some branches being associated with no peroxisomal changes, the data are shown but no statistical conclusions drawn.

**FIg. 4.**
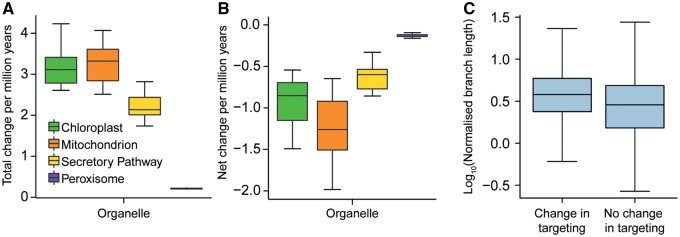
The number of changes in organellar targeting per million years for each organelle considered and the evolutionary rate of proteins undergoing a change in organellar targeting. (*A*) Nodes (*n* = 10) in the species tree for which divergence times are known were used to produce a time calibrated phylogeny. The number of changes in protein organellar targeting were then summed from the origin of the land plants (taken as 450 Ma) to each of the selected nodes and the total number of changes (gains + losses) per million years calculated. (*B*) Net change was calculated in the same way except the number of losses was subtracted from the number of gains for each branch. (*C*) Orthogroup branch lengths were normalized by their corresponding branch in the species tree. The normalized branch lengths of branches associated with and without a change in organellar targeting were compared. On average, branches (ancestral proteins) associated with a change in targeting had a higher normalized branch length, two-tailed *t*-test *P* < 0.001.

To investigate the pattern of protein gain and loss over the species tree, the number of gains and losses in protein targeting along each branch of the species tree was compared with the length of the branch, that is, the amount of molecular sequence evolution between species. There was a positive linear correlation between the amount of molecular sequence evolution and the number of changes in localization to all subcellular compartments (fig. 3*A*–*D*). Using a time-calibrated species phylogeny, it was possible to estimate that on average 3.5 (1.3 gains and 2.2 losses), 3.6 (1.2 gains and 2.4 losses), 2.4 (0.9 gains and 1.5 losses), and 0.22 (0.05 gains and 0.17 losses) changes in protein targeting to the chloroplast, mitochondrion, secretory pathway, and peroxisome occur for every million years of land plant evolution, respectively (fig. 4*A* and *B*). Thus, organellar protein content has diversified during plant evolution in proportion to molecular evolutionary distance.

### Change to Organellar Targeting Is Associated with an Increase in the Rate of Molecular Sequence Evolution

To determine whether a change in organellar targeting of an ancestral proteins was associated with an increase in the rate of molecular sequence evolution, the length of the branches in gene trees on which a change in organellar targeting occurred was compared with the length of branches on which a change in localization did not occur. To render branch lengths comparable between and across gene trees, each branch in each gene tree was normalized by the length of the corresponding branch in the species tree that was inferred from concatenated single-copy genes (see Materials and Methods). This revealed that the lengths of branches in gene trees on which a change in organellar targeting occurred are longer than those branches on which a change did not occur (*P* < 0.001, fig. 4*C*). This difference was not due to a difference in the number or phylogenetic distribution of these branches, as the same difference was also observed if the number and phylogenetic distribution of sampled branches were kept constant between the two groups (*P* < 0.001, Monte Carlo resampling). Thus, changes in organellar protein targeting are associated with an increase in gene evolutionary rate.

### Changes in Organellar Targeting Occur More Frequently Following Gene Duplication

**FIg. 5.**
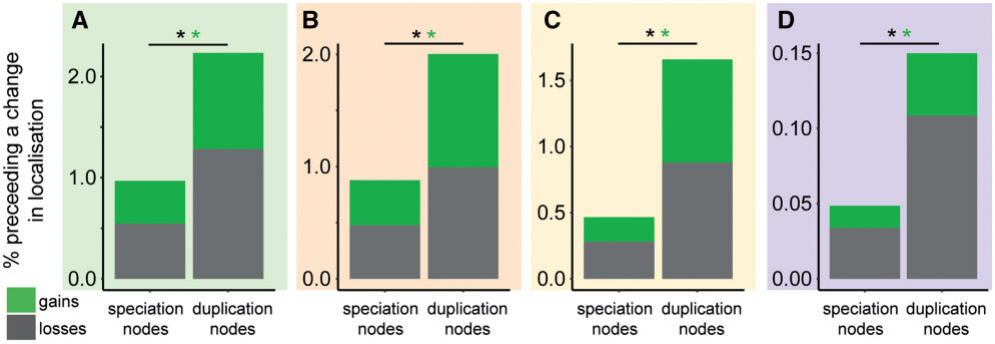
The difference in rates of change in organellar targeting following gene duplication or nonduplication (speciation) events in (*A*) the chloroplast, (*B*) the mitochondrion, (*C*) the secretory pathway, (*D*) the peroxisome. *Significant difference *P* < 0.01.

Given that changes in protein targeting require relatively drastic changes in the functional coding sequence of proteins, it was hypothesized that such changes might be more likely to be retained if they occurred to recently duplicated genes. Furthermore, it has been previously suggested that changes in protein targeting following gene duplication may be an important mechanism of duplicate gene neofunctionalization (Byun-McKay and Geeta 2007; Marques et al. 2008; McKay et al. 2009; Byun and Singh 2013). If these prior hypotheses are correct, it would be expected that changes in protein targeting would occur more frequently following gene duplication events in our data set. To test whether this phenomenon occurred, the association between gene duplication and changes in organellar targeting of proteins was investigated (see Materials and Methods). Across angiosperms, a robust set of 19,353 gene duplication events were identified and the frequency with which changes in protein targeting occurred on either of the two direct descendant child branches of each of these gene duplication events was analyzed. This revealed that there was a change in organellar targeting along one of the two immediate child branches for 1,072 (5.5%) of these gene duplication events (full data set available in the Zenodo supplementary data archive). This frequency was significantly higher than that observed for nodes that did not contain a gene duplication event in the same gene trees (2.2%, hypergeometric test, *P* < 0.01; supplementary file S3, Supplementary Material online). This phenomenon is observed whether the data set is analyzed as a whole or whether gains and losses to individual organelles are analyzed separately (fig. 5*A*–*D*). To account for any potential biases in the distribution of gene duplication events in the species phylogeny, an analogous analysis was conducted where the number and phylogenetic position of the nonduplicated nodes were randomly sampled so as to be identical to that of the identified gene duplication events (see Materials and Methods and supplementary file S1, Supplementary Material online). This revealed the same result, whereby there was a higher frequency of changes in organellar targeting following branches of the tree along which a gene duplication is predicted to have occurred, compared with those without. Thus, overall the frequency of evolving a change in organellar targeting is higher following gene duplication.

### There Is No Difference in the Frequency of Organellar Targeting Changes Following Single-Gene Duplications or Whole-Genome Duplications

**FIg. 6.**
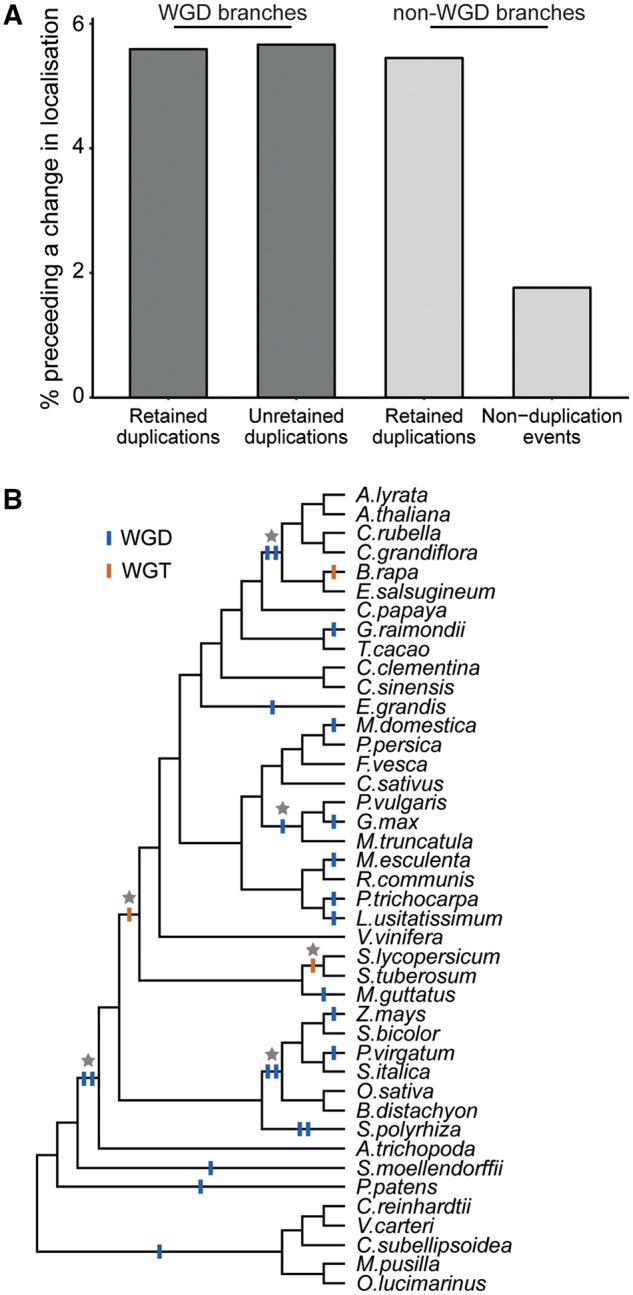
Change in organellar targeting was found to occur more frequently following gene duplication. (*A*) The number of changes in organellar targeting was significantly higher following gene duplication (hypergeometric test, *P* < 0.05). This was true for gene duplications arising from whole-genome duplication events and from single-gene processes. This increase in the rate of change of organellar targeting was also observed following gene duplication even when one of the duplicate pair was subsequently lost. (*B*) The species tree showing branches along which whole-genome duplication and triplication events are thought to have occurred (see references in supplementary file S3, Supplementary Material online). The nonterminal branches containing whole-genome duplication events used in this study are indicated with stars. Although terminal branches of the species tree were not considered in the analysis in (*A*), the presence of whole-genome duplication and triplication events have been shown for completeness.

Gene duplications can arise from single-gene processes (such as tandem duplication) or from whole-genome duplication or triplication events. To investigate whether there was an effect of duplication type of the likelihood of a protein gaining or losing an organellar targeting signal following gene duplication, all identified gene duplications were categorized into one of two sets: Set 1 comprised the cohort of gene duplications that originated on branches in the species tree on which a whole-genome duplication (or triplication) event is thought to have occurred (Jiao et al. 2011; Lee et al. 2012; Vanneste et al. 2014; Ren et al. 2018); Set 2 comprised the cohort of gene duplicates that originate on branches for which there is no associated whole-genome duplication, and thus are assumed to have arisen from single-gene processes (supplementary file S3, Supplementary Material online). Comparison of these two sets revealed that there is no difference in the likelihood of a change in protein localization following gene duplication from either single-gene or whole-genome duplications (fig. 6). The proportion of duplicates from whole-genome or single-gene events that subsequently underwent a change in organellar targeting was 5.4% and 5.6%, respectively (compared with 2.3% for nonduplication nodes) (fig. 6, supplementary file S3, Supplementary Material online).

Identification of branches in the species tree that are associated with whole-genome duplication allowed the identification of an additional independent set of “fossil gene duplicates.” These are genes which must have existed in a duplicated state for a period of time before returning to single-copy status prior to the subsequent speciation event and thus one duplicate from the pair no longer exists in any extant species in this analysis. The existence of these fossil duplicates is a corollary of the fact that a whole-genome duplication must have duplicated all of the genes, or else it was a partial genome duplication. Interestingly, this group of fossil gene duplicates also exhibited the same high rate of change in organellar targeting as duplicated genes that were retained (5.7%; fig. 6 and supplementary file S3, Supplementary Material online). This suggests that the rate of change in protein targeting is also elevated for duplicated genes where one duplicate is subsequently lost from the genome.

### Changes in the Regulatory Machinery Are the Main Changes Occurring to the Chloroplast and Mitochondrial Proteomes

**FIg. 7.**
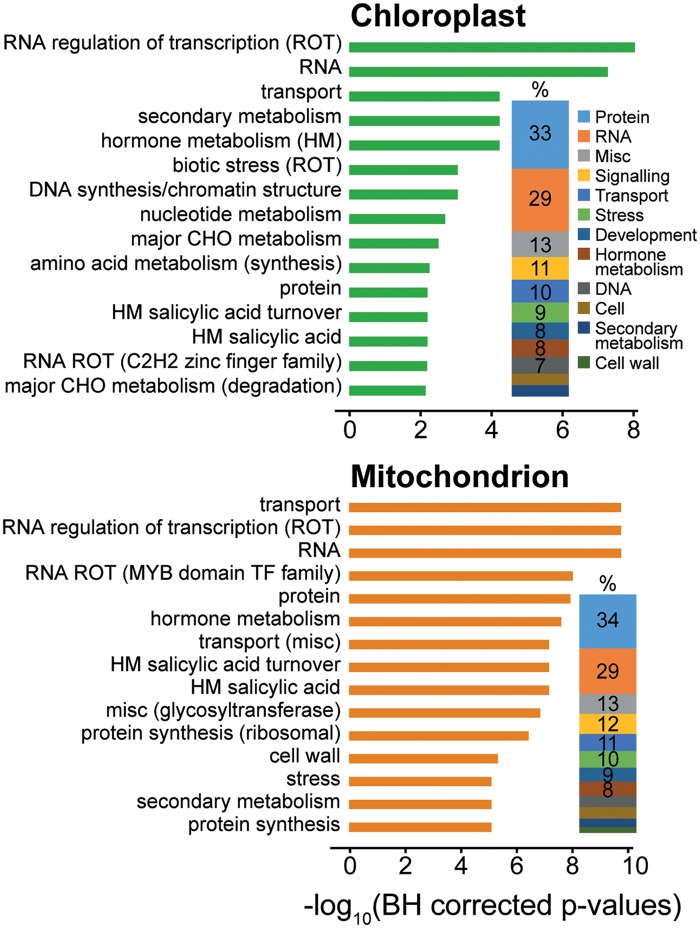
Enriched functional terms (GOMapMan) for the set of proteins that gained or lost a chloroplast or mitochondrial target peptide during the evolution of the 42 plant species in this dataset. The top 15 terms are shown for display purposes and the full data set is available in supplementary file S4, Supplementary Material online. The proportion plot next to the bar plot indicates the percentage representation of top-level functional categories encompassed by the full set of enriched functional terms.

To shed light on the functional significance of these changes in organellar protein targeting, a functional term enrichment analysis was conducted on the set of genes whose localization changed during plant evolution. For both the chloroplast and the mitochondrion, the set of genes that changed organellar targeting during evolution (when compared with the complete set of proteins predicted to be targeted to that organelle) were found to be enriched for functional terms concerning regulation, both at a transcriptional level and a posttranscriptional level (fig. 7). There was also an overrepresentation of functional terms concerning hormone production, secondary metabolism, stress, transport, and development (supplementary file S4, Supplementary Material online), with few terms related to energy metabolism. In support of this observation, among proteins gained and lost to the chloroplast there was also an overrepresentation of proteins that localize to the nucleoid, with no statistical overrepresentation of proteins that localize to other chloroplast subcompartments such as the thylakoid, envelope, or stroma (supplementary file S5, Supplementary Material online). Analogous findings were also observed for the mitochondrion (fig. 7). Thus, changes to the regulatory landscape of organelles has been the major consequence of changes in protein targeting during the evolution of chloroplasts and mitochondria in land plants.

Consistent with the lack of genetic material, functional terms associated with transcriptional regulatory processes were not observed for either the peroxisome or secretory pathway (supplementary file S4, Supplementary Material online). Instead, enriched functional terms for peroxisomal proteins were associated with metabolism (amino acid, lipid, secondary) or gluconeogenesis, whereas changes in the cohort of proteins targeted to the secretory system were associated with protein posttranslational modification, signaling, and the cell wall (supplementary file S4, Supplementary Material online).

It has previously been suggested that proteins with core organellar function are more likely to be sensitive to dosage imbalance following gene duplication. Evidence for this comes from the fact that gene families that tend to maintain single-copy status throughout plant evolution are enriched for chloroplast/mitochondrion-related function, as well as other housekeeping functions (De Smet et al. 2013; Li et al. 2016; Tasdighian et al. 2017). Concomitant with this, it is expected that proteins with these core functions will also be unlikely to undergo changes in subcellular localization. To investigate this, a functional term enrichment analysis was conducted for those orthogroups with no evidence of changes in organellar targeting during their evolution. For orthogroups with no history of changes in chloroplast and mitochondrion targeting, there was an enrichment for functional terms related to photosynthesis and mitochondrial electron transport, respectively (supplementary file S4, Supplementary Material online). There was also an enrichment for functional terms relating to DNA synthesis and chromatin structure suggesting that genes with core organellar and cellular functions are also less likely to undergo changes in organellar targeting.

## Discussion

The partitioning of diverse cohorts of proteins into organelles facilitated the evolution of complex multicellular life (Lane and Martin 2010). As a consequence, the origins and early evolution of organelles have been the subject of much research (Mast et al. 2014). The study presented here provides substantial new insight into the dynamics of organellar proteome evolution in land plants. It reveals that there has been continuous change in the nuclear-encoded proteome of organelles since plants colonized the land ∼450 Ma. Furthermore, this study uncovers a key role for gene duplication in accelerating the pace of organellar proteome evolution in plants, revealing a novel mechanism by which evolutionary changes in the nuclear genome impact on the evolution of organelles. The implications of this and the fact that changes in organellar targeting are enriched among proteins with gene regulatory functions are discussed below.

Although there has been much interest in comparison of organellar genome content between species (Palmer et al. 2000; Green 2011; Daniell et al. 2016), to date there has been little research into the diversity between the nuclear-encoded organellar proteomes of different eukaryotic species. Furthermore, although it is widely reported that orthologous genes can encode proteins with disparate subcellular localizations, there has been no investigation into the extent to which changes in organellar targeting of proteins occur during evolution. The analyses in this study suggest that there have been substantial changes to the nuclear-encoded proteome of organelles during plant evolution. At least 6,480 and 6,157 gains and losses in protein targeting were identified to have occurred to the chloroplast and mitochondrion, respectively, since the evolution of land plants. This amounts to considerable modulation of the organellar proteomic environment, far greater than that which results from changes in organellar genome content. For instance, comparison of changes in chloroplast gene content among a diversity of plants similar to that used in this study (covering 64 species) found that most angiosperm plastid genomes contain 113 different genes, and that during the evolution of these species only 62 gene loss events among 38 different genes have occurred (Jansen et al. 2007). Similar reports of genome stability among angiosperms have been made for the mitochondrion genome (Adams et al. 2002). Thus, by comparison, there has been a far greater change (2 orders of magnitude more) to the proteome of the chloroplast and mitochondrion as a result of changes in organellar targeting of nuclear-encoded proteins. This may not, however, reflect relative functional impact given that cytoplasmically encoded proteins represent a highly conserved and specialized suite of proteins that constitute the core functions of organelles. Indeed, it has previously been reported that genetic variation at organellar loci has a disproportionately large effect on phenotype compared with equivalent nuclear loci (Joseph et al. 2013; Dobler et al. 2014). Nonetheless, the findings in this study suggest that considerable modulation of organelle proteomes has occurred during plant evolution via changes in protein targeting of nuclear-encoded genes, and that such changes may be a pervasive mechanism by which genes acquire new functions. It will be interesting to know whether this phenomenon of continual change in organellar targeting of nuclear-encoded proteins occurs in other eukaryotic lineages.

Gene duplication (whether through individual duplications or whole-genome multiplications) is a recurrent theme in eukaryotic evolution (Zhang 2003; Taylor and Raes 2004; Soltis et al. 2015) and has been proposed as a major mechanism by which new genetic material is generated (reviewed in Long et al. 2003; Conant and Wolfe 2008; Magadum et al. 2013; Panchy et al. 2016). It has previously been suggested that gene duplication might facilitate changes in organellar protein targeting due to that fact that, in some instances, gene duplication leads to genetic redundancy and a relaxation of purifying selection on one or both gene copies (Byun-McKay and Geeta 2007; Marques et al. 2008; McKay et al. 2009; Byun and Singh 2013). Under these conditions, the accumulation of the genetic changes necessary for the evolution of a new target signal (or its loss) may be facilitated. Such a change could then be fixed by either drift (in the case for neutral or weekly deleterious alleles) or selection (for advantageous alleles). The findings presented in this study corroborate this hypothesis, with changes in organellar targeting more likely to occur (or be retained) following gene duplication. As gene duplication facilitates changes in organellar targeting, it is thus a key facilitator of organellar evolution.

Although we observed an elevated rate of change in organellar targeting following gene duplication, we did not observe a difference in the rate of change following individual gene and whole-genome duplication events. The lack of difference here is perhaps surprising given there have been repeated observations that there are biases in the types of genes that are retained following these distinct duplication mechanisms (Conant et al. 2014; Freeling et al. 2015; Wendel et al. 2016). Specifically, interconnected genes, such as those that form multiprotein complexes or those that encode genes with gene regulatory functions, are preferentially retained following whole-genome duplication, whereas the same sets of genes tend not to be retained following individual gene duplications (Maere et al. 2005; Blomme et al. 2006; Freeling 2009; Tasdighian et al. 2017; Liang and Schnable 2018; Wendel et al. 2018). The reason for this is that natural selection acts to maintain stoichiometry and/or gene dosage. Following whole-genome duplications, the duplication of all highly interconnected genes means that the loss of any one copy will be selected against in order to prevent dosage balance perturbations. Concomitantly, individual duplications of the same genes are expected to revert to single copy for the same reason. A priori, one would expect that selective pressure to maintain stoichiometry and/or gene dosage would also influence whether a change in organellar targeting is retained or lost. Moreover, one would expect a low rate of change in organellar targeting in genes that are dosage sensitive as a change in the localization of a gene product is akin to loss of the gene in its ancestral location. Among proteins that had undergone changes in organellar targeting during evolution, there was no particular enrichment for proteins that form multiprotein complexes, but there was an enrichment for gene regulatory functions (e.g., transcription factors). Therefore, the hypothesis that dosage sensitive genes are not likely to undergo changes in organellar targeting does not hold true for regulatory genes. It has been previously suggested, however, that regulatory proteins may be retained through other mechanisms than dosage sensitivity following whole-genome duplication based on the divergent pattern of gene expression between paralogs of regulatory proteins. (Blanc and Wolfe 2004; Tasdighian et al. 2017). Another set of dosage-sensitive genes are those that are resistant to gene duplication and consistently return to single-copy status. Among such gene families is enrichment for functions in genome integrity and organelle function (Blanc and Wolfe 2004; Li et al. 2016). Again, it is expected that these genes will be resistant to changes in organellar targeting. Interrogation of the functions of genes that did or did not evolve changes in organellar targeting supported this hypothesis. Specifically, we observed enrichment for functional terms related to dosage-sensitive genes involved in core organellar processes (i.e., photosynthesis or mitochondrial electron transport/ATP synthesis) among the proteins which have never undergone changes in organellar targeting. Thus, although there is no difference in the rate of organellar targeting change following individual or whole-genome duplication events, the dosage sensitivity of a gene may affect its predisposition to changes in localization.

## Conclusions

The results from this study present a holistic picture of a previously unstudied mechanism of organelle evolution. Moreover, this is the first study to quantify the extent to which changes in subcellular localization of proteins have occurred during the evolution of a major group of eukaryotes. By using genomic data from a sampling of organisms that span the breadth of the Plant Kingdom, what has emerged is a picture of a dynamic organelle proteome which has been shaped by continual changes in the subcellular targeting of proteins, substantially altering the regulatory landscape of these organelles. Moreover, it has revealed a novel way in which gene and whole-genome duplication play a role in facilitating organellar evolution.

## Materials and Methods

### Problem Definition and Approach

In this study, we aimed to identify the changes that have occurred to plant-cell organellar proteomes following the adaptation of plants to land. To do this, a phylogenomics approach was taken to predict when changes in protein targeting occurred during the evolution of different gene families. Species-tree-reconciled gene trees were inferred from genome data of 42 diverse plant species allowing us to map gains and losses in organellar protein targeting across the species phylogeny. These gains and losses were then interrogated to answer questions about the nature of organelle proteome evolution in the Plant Kingdom and the molecular mechanisms that drive changes in protein targeting. Full details of each step of this approach, as well as the complete data set and all scripts required to repeat the analysis are described and provided below.

### Construction of Orthogroups and Inference of Species-Tree-Reconciled Gene Trees

Protein sequences corresponding to the primary transcripts of 42 fully sequenced plant species were obtained from Phytozome v10 (Goodstein et al. 2012). OrthoFinder (Emms and Kelly 2015, 2019) and MAFFT-LINSI (Katoh and Standley 2013) were used to infer orthologous gene groups (orthogroups) and their multiple-sequence alignments, respectively. Only orthogroups with at least four genes and representation from more than one species were taken forward for analysis.

We used PHYLDOG (Boussau et al. 2013) to simultaneously infer orthogroup gene trees and reconcile these gene trees to the species tree. PHYLDOG takes a joint likelihood approach to infer gene trees, using both the multiple-sequence alignment and the known species tree. This reconciliation method was used to minimize the effects of gene-tree inference error and weakly supported partitions in gene trees. Moreover, PHYLDOG has previously been shown to improve gene-tree reconstruction compared with methods that do not take the species tree into account (Szllosi et al. 2015). In all cases, orthogroup multiple-sequence alignments were trimmed to remove columns containing more than 66% gap characters prior to PHYLDOG tree inference using the “LG08” model of sequence evolution. Some of the largest orthogroups were too large to be analyzed directly with PHYLDOG (the largest orthogroup contained 12,148 genes). Manual inspection revealed that these large orthogroups were not single orthogroups, but instead were fusions of multiple orthogroups originating from a gene duplication event that preceded the diversification of the species in the analysis. Thus, to enable the analysis of these data, the gene trees for these fused orthogroups were split into correctly circumscribed individual orthogroups at the ancient duplication node by a process of tree inference and gene tree–species tree reconciliation. Each of these disentangled orthogroups was then analyzed by PHYLDOG as described above.

To run PHYLDOG on the multiple-sequence alignments described above requires a species tree with branch lengths as input. Here, the topology of the species tree was derived from the angiosperm phylogeny working group (Stevens 2001). However, this tree did not contain branch lengths. Thus to infer branch lengths for this species tree, we constructed a concatenated multiple sequence alignment of all single-copy gene orthogroups that contained ≥75% of the species (*n* = 1,230). This concatenated alignment was subject to phylogenetic tree inference with the topology constrained to the known species tree using FastTree (FastTree -gamma -nome -mllen -intree SpeciesTree.txt ConcatenatedAlignment.al > SpeciesTree_constrained.txt) (Price et al. 2010).

To provide a methodologically independent control, and mitigate against any potential overfitting caused by use of the PHYLDOG method, we also carried out the complete analysis on unreconciled gene trees that were inferred directly from the multiple sequence alignments using IQ-TREE with the settings –m TEST to automatically select the best-fitting model of sequence evolution for each gene tree inference (Nguyen et al. 2015). Here, individual gene-tree branches were mapped to branches of the species tree using a heuristic method of last common ancestor identification as described previously (Swenson et al. 2012). Additional information is also provided in supplementary file S1, Supplementary Material online.

### Prediction of Organellar-Targeted Proteins

Of the 42 species included in this study, 37 comprise land plants and five comprise green algae. For each species, we identified the set of proteins predicted to contain a chloroplast transit peptide, mitochondrial target peptide, secretory signal peptide, or the peroxisomal targeting signals 1 and 2 (PTS1 and PTS2). For the land plant species, chloroplast transit peptides, mitochondrial target peptides, and signal peptides were predicted using TargetP 1.1 (Emanuelsson et al. 2000) in plant mode with default cutoffs. For the five algal species (*Ostreococcus lucimarinus, Micromonas pusilla, Coccomyxa subellipsoidea, Volvox carteri, Chlamydomonas reinhardtii*), this prediction was carried out with PredAlgo (Tardif et al. 2012) using its default cutoffs. In cases where an amino acid sequence did not meet the minimum length requirement for PredAlgo prediction, the TargetP prediction was taken instead.

The prediction of peroxisomal proteins was carried out by searching for the canonical plant PTS1 and PTS2 (Reumann 2004). Here, a protein sequence was classified as having a PTS1 if it had any one of the nine different C-terminal tripeptide sequences: SRL, SRM, SRI, ARL, ARM, PRL, SKL, SKM, AKL. A protein sequence was classified as having a PTS2 peroxisome targeting sequence if it contained either of the PTS2 peptide sequences (R[LI]X_5_HL) in the N-terminus region of the protein (residues 1–30). TargetP does not take into account cases of dual localization; however, if a protein was found to have a PTS and a positive TargetP localization, it was labeled as dual peroxisomal localized.

TargetP was selected as the main target signal predictor as it performs well in benchmarks (Klee and Ellis 2005), is available to download, and has a “plant” mode based on a neural network trained on plant data. To provide additional support for the findings presented in this study, we also ran the complete analysis using two alternative subcellular localization predictors which take contrasting approaches to target signal prediction—WoLF PSORT (Horton et al. 2007) and iPSORT (Bannai et al. 2002). The results from these independent analyses fully replicate and support the findings presented in the main text, and are provided in supplementary file S1, Supplementary Material online.

### Ancestral Character Estimation of Subcellular Targeting

Ancestral gains and losses of protein targeting were identified in orthogroups using maximum-likelihood ancestral character estimation (ACE). For each protein, the presence or absence of a particular organellar target signal was treated as binary trait data and the leaves (i.e., genes) of the orthogroup trees assigned “1” or “0” accordingly. Here, each type of target signal was considered separately and each orthogroup tree analyzed independently. The presence or absence of a target signal in ancestral protein sequences represented by each internal branch of an orthogroup tree was inferred using the “ACE” function in the R package ape 5.2 (Paradis et al. 2004) using the “all rates different” model for discrete data. The all rates different model was selected as the transition probabilities between states (i.e., presence/absence of target signals) are unknown and cannot be assumed to be equal. Furthermore, an “all rates different” model performed better on average than a “equal rates” model as assessed by a chi-squared log likelihood ratio test across all trees.

ACE was used to infer the probability (between 0 and 1) that an ancestral protein sequence (represented by an internal branch in an orthogroup tree) had a specific organellar targeting signal. To identify changes in protein targeting in orthogroup trees, we used a winner takes all approach whereby branches with an ACE score of ≥0.5 were assigned as organellar-targeted proteins and branches with scores of <0.5 were assigned as nonorganellar-targeted proteins. Further processing and filtration were carried out as described below.

### Identifying Changes in the Subcellular Localization of a Protein during Evolution

The ACE data were analyzed to identify when changes in organellar targeting occurred during the evolution of an orthogroup. Losses in organellar targeting were identified when there was a transition from a targeted state to a nontargeted state on immediately consecutive branches in the gene tree, and vice versa for a gain. As ACE is expected to be sensitive to targeting prediction error or gene tree error, a stringent filter was imposed for the identification of gains and losses. This filter required that >75% of the genes descendant from the branch on which the change is estimated to have occurred retain the changed subcellular localization state, and >75% of genes descendant from the sister branch maintain the ancestral state. For example, consider an internal bipartition within a gene tree that has two descendant sister branches X and Y. If a gain in chloroplast targeting is predicted to occur on branch X, then >75% of the genes that are descendant from branch X must contain a predicted chloroplast targeting signal, whereas >75% of the genes that are descendant from branch Y must not contain a predicted chloroplast targeting signal. Only if both these criteria are met would a change in subcellular localization be assigned to branch X in the orthogroup tree. This requirement meant that inference about the predicted localization of an ancestral protein was always informed by the predicted localization of three or more extant genes. Furthermore, it was also required that sequences from two or more species must subtend any branch under consideration. A worked example of the application of this filter is provided in supplementary file S1, Supplementary Material online. Only the changes in organellar targeting that passed this filter were used in the subsequent analyses presented in this study. The branches within orthogroup trees on which these changes occurred were mapped to branches in the species tree using the gene tree–species tree reconciliation provided by PHYLDOG. This enabled the number of gains and losses in protein targeting to each of the four organelles to be tallied for each branch of the species tree.

It is possible that gene loss or incomplete genome annotation can lead to uncertainty in the mapping of gene-tree branches of the species tree. In certain instances, the absence of a gene from a gene tree can cause a branch in the gene tree to map to two consecutive branches in the species tree (example is provided in supplementary file S1, Supplementary Material online). In these instances, PHYLDOG maps these gene-tree branches to the most recent branch in the species tree using a most recent common ancestor approach (Boussau et al. 2013). In total, just 21% of gains and 17% of losses occur on gene-tree branches that (either through either real gene loss or erroneously missing gene models) correspond to two consecutive branches of the species tree. To investigate whether the use of an alternative mapping approach would affect the overall result, a separate analysis was conducted. This time, when a change in organellar targeting occurred on a gene-tree branch that maps to multiple consecutive branches in the species tree, the gain or loss was distributed equally between those branches in the species tree (rather than just the most recent branch). The altered placement of the 21% of gains and 17% of losses did not change the global pattern of gain and loss that is observed across all orthogroups (supplementary file S1, Supplementary Material online).

### Analysis of Molecular Sequence Evolution Rate and the Evolutionary Rate of Change in Organellar Targeting

The rate of molecular sequence evolution for each orthogroup-tree branch was calculated as the length of the branch length (substitutions per site) divided by the length of the corresponding branch in the species tree (also substitutions per site). This normalization was conducted to allow relative rates of molecular sequence evolution (relative to the consensus rate estimated from a concatenated multiple-sequence alignment of single-copy genes) to be compared across branches both within and between orthogroup gene trees. To mitigate against error introduced by ambiguity in mapping (as discussed above) and the effects of gene duplication, we only calculated this normalized rate of molecular sequence evolution for branches which could be uniquely placed in the species tree and which had no evidence of gene duplication. For all qualifying branches, the normalized rate of molecular sequence evolution for those branches associated with a change in organellar targeting was compared with the analogous rate for branches that had no evidence for a change in organellar targeting. To further mitigate against potential biases arising from differences in the phylogenetic distribution of these two sets of gene-tree branches, we used a random sampling approach whereby the number and distribution of branches sampled for both sets were identical. This random sampling process was repeated 1,000 times to obtain the Monte Carlo *P*-value that is reported in the main text.

### Incorporation of Divergence Time Data

To estimate the average rate at which proteins have gained or lost organellar target signals during the evolution of land plants, ten nodes were selected from the species tree for which a divergence time is known. The number of gains and losses in targeting to each organelle was then summed for the branches between the node at the base of the land plants (taken as 450 Ma [Morris et al. 2018]) and each of these ten dated-nodes thereby allowing the number of changes per million years to be calculated (supplementary file S2, Supplementary Material online). It should be noted, however, that the ten nodes that were selected all share at least part of a common path of evolution and are therefore nonindependent. For this reason, the full range of the estimates is shown as box plots but confidence intervals are not provided.

### Identification of Changes Following Gene Duplication and Speciation Events

To investigate whether changes in organellar protein targeting occur more frequently following gene duplication events or nonduplication (speciation) events, it was necessary to identify nodes which correspond to these events in each orthogroup tree. To prevent tree inference error from influencing the results, a stringent filter was applied to enable identification of high-confidence gene duplication nodes and speciation nodes in each orthogroup tree. High-confidence gene duplication nodes were defined as nodes for which a gene duplication event was retained in all descendant species of both child branches subtending the duplication event. Similarly, a high-confidence speciation node was selected as a node which has no evidence for gene duplication and from which there was no subsequent gene loss in any of the descendant species. In both cases (duplication and speciation nodes), complete retention of all genes in all descendant species is required and thus the gene sets can be considered equivalent. A corollary of this stringent selection criterion is that the branches which pass this filter are also unambiguously placed within the species tree.

The occurrence of change in organellar targeting on the single branch immediately following these gene duplication nodes and speciation nodes (i.e., along the two direct child branches subtending the node) was analyzed. Changes in more distant branches (i.e., grandchild nodes or great grandchild notes, etc.) were not considered in this analysis. Thus, only changes in localization that occurred before the next speciation event or gene duplication event were analyzed. The number of descendant branches for gene duplication nodes and speciation nodes is the same, *n* = 2, that is, every branch in every tree has two descendant branches irrespective of whether it is a gene duplication branch or a speciation branch.

To mitigate against potential biases arising from differences in the phylogenetic distribution of these two sets of gene-tree branches (the gene duplication set and the nonduplication set), a random sampling approach was also conducted whereby the number and phylogenetic distribution of branches sampled for both sets were identical. This random sampling process was repeated 1,000 times to obtain the Monte Carlo *P*-value that is reported in the main text. The results from this analysis can also be found in supplementary file S1, Supplementary Material online.

### Functional Term Enrichment Analysis

Orthogroups were assigned MapMan terms and subchloroplast localization terms by inheriting the terms associated with the genes found within them. MapMan terms were taken from the GoMapMan webpage (Ramšak et al. 2014) and subchloroplast terms assigned using the hierarchical structure provided on the Plant Protein Database (Sun et al. 2009) using only experimentally validated proteins (see supplementary file S5, Supplementary Material online, for the PPDB list used at time of writing). To test for enrichment, the hypergeometric test was performed and *P*-values corrected for multiple testing using the Benjamini–Hochberg correction (see supplementary file S4, Supplementary Material online, for MapMan results and supplementary file S5, Supplementary Material online, for PPDB). The aim was to identify functional enrichment among orthogroups whose proteins are differentially localized. To avoid simply identifying functional terms that are enriched in organelle-targeted gene families, the background sample for this test was orthogroups with at least one predicted organelle-targeted protein. Significantly enriched functional annotation terms were those with a corrected *P*-value of ≤ 0.01.

### Availability of Data and Material

All data used and generated in this study are available in the Zenodo research data archive at the following address: https://doi.org/10.5281/zenodo.1414180. This archive contains the full set of sequences, accession numbers, predicted localization data, orthogroups, and PHYLDOG-reconciled gene trees for each orthogroup. The archive also contains a data file detailing all the gene duplication events and changes in protein targeting events that were inferred for each orthogroup. A GitHub repository containing all relevant code necessary to repeat the analysis is available at https://github.com/RonaCostello/charting-organelle-protome-evolution.

## Supplementary Material

msz275-Supplementary_Data
