# Supplementary material for: Gene Duplication Accelerates the Pace of Protein Gain and Loss from Plant Organelles
Source: Mol Biol Evol. 2019 Nov 21;37(4):969–81. doi: 10.1093/molbev/msz275 (PMC7086175; doi:10.1093/molbev/msz275)
Supplement: msz275-Supplementary_Data [file msz275-supplementary_data.zip › msz275-Suppl_Data/Supplemental File S1.pdf]

Supplemental File S1

Justification for using species-tree reconciled gene-trees and comparison with a non-reconciled method

It is expected that all gene trees contain tree inference error, otherwise a correct species tree could be accurately inferred from a gene tree of any single copy gene. The presence of this inference error has been repeatedly shown across all domains of life and in an exemplar analysis of 1030 gene trees of one-to-one orthologs from 23 fungi species, all 1030 gene trees were topologically distinct from each other and from the species tree (Salichos and Rokas 2013). Thus, gene tree inference error is abundant and will negatively impact on any analysis that relies upon the topology of such trees. To minimize the impact of this inference error in this study, a phylogenetic method that takes a joint likelihood approach to infer gene trees was used. This method incorporates both the multiple sequence alignment of a gene family and the known species tree to infer gene-trees. This approach was taken as it directly addresses the issue of gene tree inference error and improves the accuracy of gene tree reconstruction (Szllosi et al. 2015). The results of this analysis are presented in the main text and figures.

However, to provide a comparison using a different phylogenetic method that does not incorporate the known species tree in the gene tree inference step, the complete set of gene trees was also inferred from multiple sequence alignments using IQ-TREE (Nguyen et al. 2015). As these gene trees are not reconciled to a species tree, it is unknown which branches in the gene trees correspond to specific branches in the species tree. To enable a collective analysis of these un-reconciled gene trees, gene-tree branches were mapped to branches in the species-tree using PHYLDOG (Boussau et al. 2013) which takes a heuristic method of most recent ancestor identification (Swenson et al. 2012). Following this, ancestral state estimation was conducted as described in the main text to identify changes in organellar protein targeting.

Fig. S1 summarizes the results from repeating the main analyses of this study using these unreconciled trees. It also shows a comparison of the magnitude of relocalisations identified across the species tree compared to the PHYLDOG method. Concomitant with the finding in the main paper, we identified

organellar targeting gains and losses across the species tree, and the number of changes was positively correlated with branch length (Fig. S1a). While there is some variation in the total number of gains and losses identified using these two datasets, the overall trend (i.e. the amount of change for each species tree branch) are very tightly correlated (Fig. S1b). We also found that, as with the results from the PHYLDOG dataset, the frequency of evolving a change in organellar targeting to be higher following gene duplication events compared to non-duplication (speciation) events. Thus, whether reconciled or non-reconciled trees are used the overall picture of protein gain and loss from these organelles remains the same.

Justification for the use of a 75% retention cutoff in the identification of changes in organellar targeting of ancestral proteins

Fig. 2 in the main text of this paper shows the species tree with changes in organellar targeting of ancestral protein sequences mapped to it. These numbers were obtained following filtration of changes in protein targeting to those with a protein localisation retention score of 75%. This filter required that >=75% of the extant genes descendant from a branch on which a change is thought to have occurred must be retain the changed organellar targeting state, while 75% of genes descendant from the sister branch must maintain the ancestral state. Put simply, this retention score describes the proportion of genes subtending a branch along which a change in protein targeting occurred whose predicted localisation supports the change.

As a worked example of this, Fig. S2 shows the orthogroup tree for orthogroup OG0002487. This is the orthogroup containing the enzyme 2-phosphoglycolate phosphatase (PGLP), mostly commonly known to be the first enzyme in the photorespiratory pathway. In *Arabidopsis* there are two genes in this orthogroup, the canonical chloroplast-localised PGLP1 gene with photorespiratory activity, and a second gene called PGLP2 which has been shown to be localised instead to the cytosol and currently has unknown function (Schwarte and Bauwe 2007). As in *Arabidopsis*, most plants have at least one copy of both PGLP1 and PGLP2. It is unknown however if the presence of one chloroplast and one cytosolic PGLP is common across plants. In Fig. S2 the orthogroup tree for PGLP is shown. At each node in the

tree, the likelihood that the ancestral protein represented by that node has a chloroplast transit peptide is given (green pie chart). These likelihood scores were computed using ancestral state estimation (ACE) given the predicted localisation of the extant PGLP genes. A winner-takes all approach was taken such that nodes are denoted as ‘chloroplast targeted’ if their likelihood of being so is >50%. By this approach we can identify three occurrences of a change in chloroplast targeting which are indicated by the asterisks in the figure. However, only one of these changes has a 75% localisation retention in the extant genes (indicated by a red asterisk) and so is the only one we’d consider in further analysis.

Further inspection of this orthogroup tree reveals that there exists a cytosolic PGLP and a chloroplastic PGLP in the majority of angiosperms considered. These two copies also appear to have arisen via a gene duplication at the base of the angiosperms after which one copy lost a chloroplast transit peptide. This example also demonstrates that after a change in organellar targeting occurs, it possible that further changes might happen at a later time point in the tree. It is for this reason that we chose a relaxed retention score of 75%. A score of 100% would have made identification of changes in organellar targeting at deeper nodes in orthogroup trees difficult due to the increased chance that there would be further changes in targeting as you move through the tree from the node to the tips. The fact that some genes may undergo multiple rounds of changes in organellar targeting will inevitably make resolving those changes more difficult. We hope however that by using a 75% retention score we apply enough stringency to filter for what appear to be well supported changes in protein targeting (like that identified for PGLP) while providing some flexibility to minor prediction errors and multiple rounds of protein re-targeting.

In selection of a suitable protein prediction retention score we filtered our ACE dataset of changes in organellar targeting using a range of retention scores. As expected, the number of changes identified for each branch of the species tree increased as the cut-off was relaxed from 100 to 0 (Fig. S3). For the majority of nodes in the species tree, we found that there was not a large difference in the number of changes in organellar targeting identified using a retention score of 100% and 75%. We found that the deeper nodes in the species tree were the only ones really affected by a relaxation in the retention score,

with more changes in organellar targeting being identified. This is was as expected given the logic set out in the previous paragraph. Further relaxation of the cut-off (50% and 25%) resulted in far more changes being identified for all nodes (Fig. S3). The Zenodo data archive made available as part of this study details all identified organelle targeting changes in all orthogroups and their accompanied retention scores in both descendant lineages so that researchers can reanalyse this data at any level of cut-off they desire.

Re-analysis of main findings using other organelle target signal prediction tools than TargetP

When this study was conceived, we looked for a subcellular localization prediction tool that could predict proteins of multiple organelles, could be run with large amounts of data (1,143,241 protein sequences) and was optimized for plants (i.e. had a plant training set for any machine learning algorithm). TargetP fitted these requirements and is used extensively by the scientific community, with over 4000 citations of its method in the literature. There are however several other tools available. The subcellular localization database for Arabidopsis proteins (SUBA) provides a ranked list of several such popular predictors (found at http://suba.live/img/predictor_accuracy.png).

To investigate whether the findings presented in the main paper of this study can be replicated using different localization predictors, we repeated the main analyses using two additional predictors from the SUBA list. Here we choose WoLF PSORT (Horton et al. 2007) and iPSORT (Bannai et al. 2002) which both employ different strategies in the prediction of subcellular proteins to TargetP. iPSORT implements a k-nearest neighbour machine classifier for a variety of protein sequence features, while WoLF PSORT relies on a set of relatively-simple predefined rules to identify target peptides. These methods contrast to TargetP’s algorithm which was trained using a neural network.

WoLF PSORT and iPSORT were used to construct two independent datasets of chloroplast and mitochondrion proteins from the same protein sequence data used in the main paper. Quirks that arose through the use of these new predictors and should be mentioned are as follows: 1) WoLF PSORT only accepts protein sequences with a length >30, all protein sequences shorter than this were labelled as

'other'. 2) WoLF PSORT detected 13 dual localized proteins in the dataset. These proteins were labelled as both chloroplastic and mitochondrial in our dataset. 3) WoLF PSORT assigns a protein sequence a score for various localizations and the highest scoring compartment was used in the assignment of protein localization (winner takes all). As with the TargetP analysis, PredAlgo (Tardif et al. 2012) was used for the prediction of algal proteins. We also enforced the same 75% retention score to the identification of ancestral changes in protein targeting.

Using the predictions made by both these tools, the main analyses of this study were repeated. Fig. S4 (a & c) replicates Fig. 3 of the main text showing the relationship between species tree branch length (amino acid substitutions per site) and the number of changes in organellar targeting identified for that branch. Here we found that, as with the TargetP dataset, there was a positive linear correlation between the amount of molecular sequence evolution and the number of identified organellar targeting changes. For both datasets we also found targeting changes to occur more frequently following gene duplication events compared to non-duplication events (Fig. S4 (b & d)). This replicated the finding when TargetP was used, as represented by Fig. 5 in the main paper.

Mapping uncertainty in the placement of retargeting events

In order to map changes in organellar targeting identified in orthogroup gene trees to branches in the species tree it was necessary to reconcile each orthogroup tree branch to the species tree phylogeny. This mapping was done using a most recent common ancestor approach (MRCA) employed by PHYLDOG (Boussau et al. 2013), Fig. S5a. In some cases, the absence of a gene from a particular clade (either through gene loss or incomplete genome annotation) can lead to uncertainty in the mapping of gene tree branches to the species tree. An example of this is shown in Fig. S5b. In these instances, MRCA mapping results in changes in organellar targeting to be mapped to the most recent branch in the species tree.

The number of re-targeting events mapped to the species tree in Fig. 2 of the main text (also shown in Fig. S6a) that were affected by this uncertainty in gene-tree to species-tree mapping was investigated. For each branch of the species tree, we identified the number of changes in organellar targeting that

were mapped to it but that could have also occurred on the consecutive branch above (i.e. ambiguous mapping). The percentage of changes in organellar targeting affected for each branch of the species tree in shown in Fig. S6b. In total, 21% of gains and 17% of losses occur on gene tree branches that correspond to multiple branches of the species tree. The results of Fig. S6b show that some branches of the species tree are more affected by this uncertainty in mapping than others. For example, in less than 50% of the changes mapped to the ancestral branch of *M. domestica*, *P. persica* and *F. vesca* could be unambiguously placed on that branch. This susceptibility is likely caused by the fact that there is only one species (*C. sativus)* in the sister clade to this branch and a single missing gene from this species could result in mapping uncertainty. If there were two species in the sister clade then both species would have to be missing the gene for the same uncertainty to arise (which is more unlikely).

An alternative approach to mapping changes in organellar targeting to the species tree involves splitting changes across the branches of the species tree on which they could have occurred. An example of this 'proportional mapping' is given in Fig. S5b. This time, when a change in targeting occurs on a gene-tree branch that maps to multiple consecutive branches in the species tree, the gain or loss is distributed equally between those branches in the species tree (rather than just the most recent branch). The placement of gains and losses on the species tree using this proportional mapping approach was investigated and is shown in Fig. S6c. Overall more gains and losses were identified using this mapping approach due to a proportion of changes previously mapped to terminal branches now being represented on internal branches of the tree. The number of gains and losses identified for each branch of the species tree by these two mapping approaches (MRCA and proportional mapping) was found to be very tightly correlated (Fig. S6d). We therefore do not think that the choice of mapping approach greatly affects the global picture of gain and loss across the species tree. MRCA approach is perhaps more appropriate given that many missing genes represent true gene loss and so mapping a change in the ancestor of that gene is not biologically relevant given that the gene was subsequently lost. It is impossible to say whether a gene is missing due true gene loss or incomplete genome annotation and therefore given the data we have, a MRCA makes the most appropriate estimate of when a change in targeting might have occurred.

Monte Carlo random sampling of speciation nodes to compare like-for-like with duplication nodes across the species tree

In this work, the set of branches in gene trees that correspond to gene duplication events and a corresponding set of control branches, for which no evidence of gene duplication exists, were identified. For each set, the proportion of branches that are followed immediately by a change in organellar targeting (on either of the two direct child branches) are then compared. The results presented in the main text show that the proportion of nodes followed by a change in targeting is higher following a gene duplication event than a non-duplication event. However, the size of both sets are not equal, and their distribution on the species tree differs (Supplemental File 4). To exclude the possibility that this finding was a result of the difference in species tree distribution of these sets, a resampling approach was taken. To do this, control (non-duplication) branches were randomly sampled with replacement such that an identical number of branches to the number of gene duplication branches were sampled for each branch in the species tree. The proportion of branches in this randomly sampled control set that were followed immediately by a change in subcellular targeting was then evaluated. This process was repeated 1000 times to obtain a Monte Carlo p-value, and the results are presented in Fig. S7. In all samples we found the proportion of non-duplication branches followed by a relocalisation to be less than that of the duplication branches, corroborating the finding in the main text. It should be noted here that branches in the species tree on which whole genome duplication events are known to have occurred were omitted from this analysis as the control branches mapped to these branches represent lost (or fossil) gene duplications and are thus not valid controls. Moreover, the results from the main text show that this set of fossil duplicates also has an elevated frequency of being followed by a change in organellar-targeting.

Figures

Figure S1

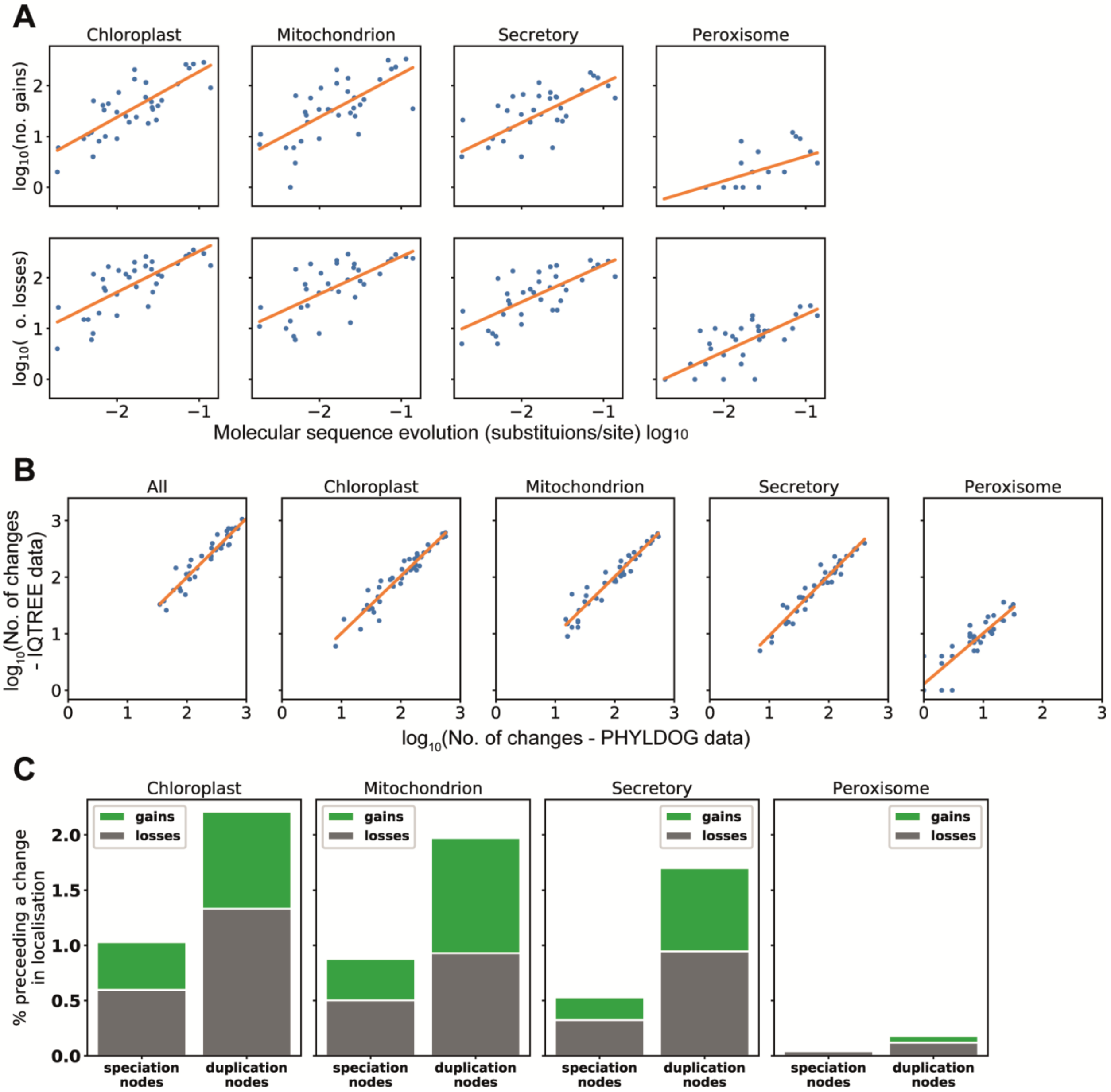

Figure S1. Comparison of orthogroup inference method (PHYLDOG vs IQTREE) in the identification of ancestral changes in organellar-targeting. A) When trees are inferred with IQTREE there is a positive correlation between species tree branch length (amino acid substitutions per site) and the number of gains and losses in organellar targeting mapped to the branch. Chloroplast (R^2 = 0.79,

0.75), mitochondrion (R^2 = 0.70, 0.68), secretory pathway (R^2 = 0.66, 0.72) and peroxisome (R^2 = 0.67, 0.75). All correlations $p < 0.001$. **B)** Across branches in the species tree, there is a very tight correlation between the number of changes in organellar targeting identified from IQTREE and PHYLDOG orthogroup trees (R^2 values being >0.9). For each branch of the species tree the number of gains and losses to each organelle were summed. **C)** Changes in organellar targeting identified in IQTREE-inferred orthogroup trees were found to occur more frequently following gene duplication ($p<0.001$, hypergeometric test, for gains and losses to each organelle).. This replicates the results found when orthogroup trees were inferred with PHYLDOG.

Figure S2

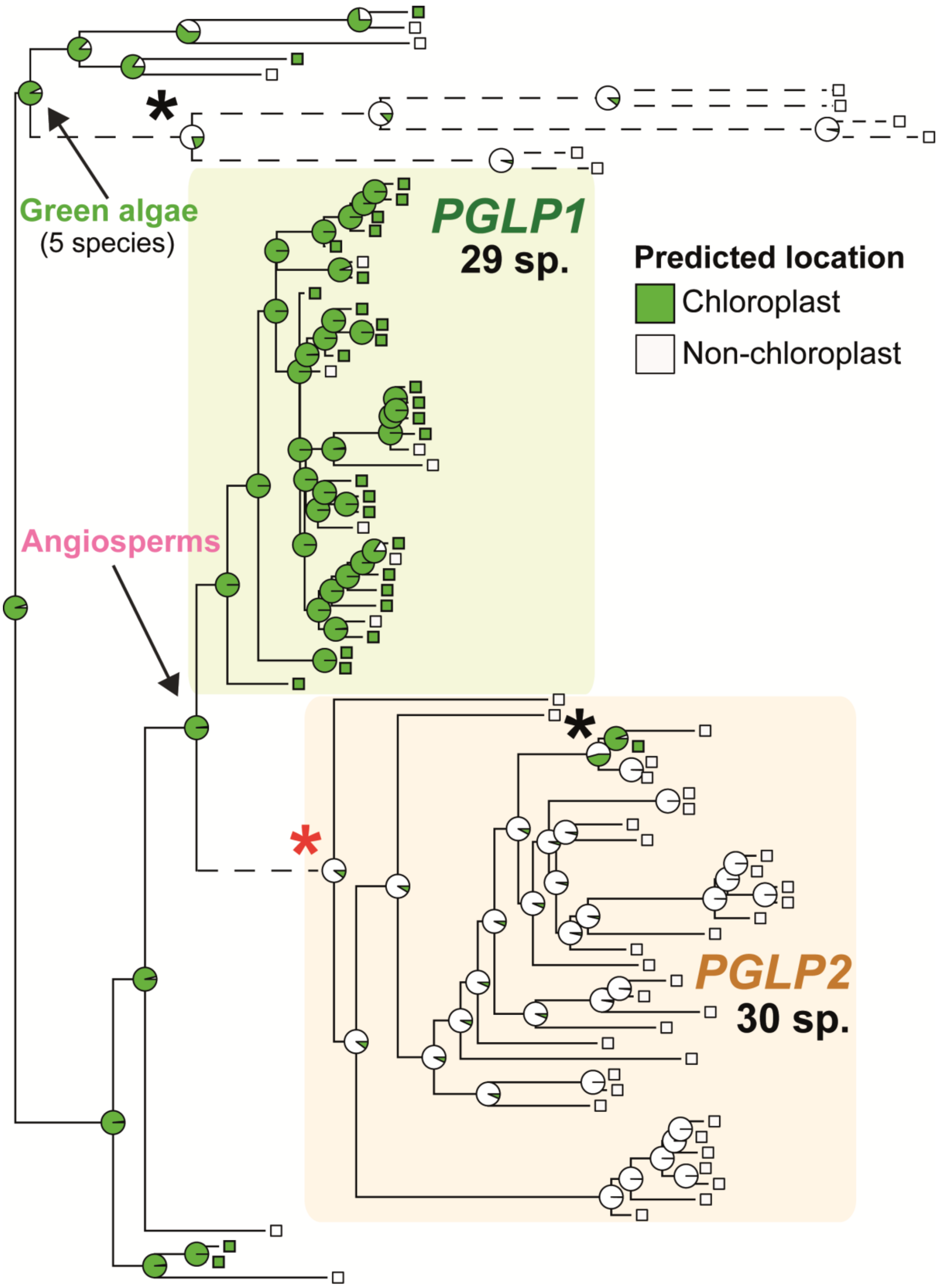

Figure S2. Orthogroup tree showing changes in chloroplast targeting during the evolution of 2-phosphoglycolate phosphatase (PGLP). Ancestral character estimation likelihoods for chloroplast

targeting of protein represented by internal nodes of the tree. The likelihood that a protein was chloroplast localised is given by the green portion of each pie chart. Boxes at the leaf nodes of the tree represent the predicted localisation of the gene at that leaf (gene names not shown).

Figure S3

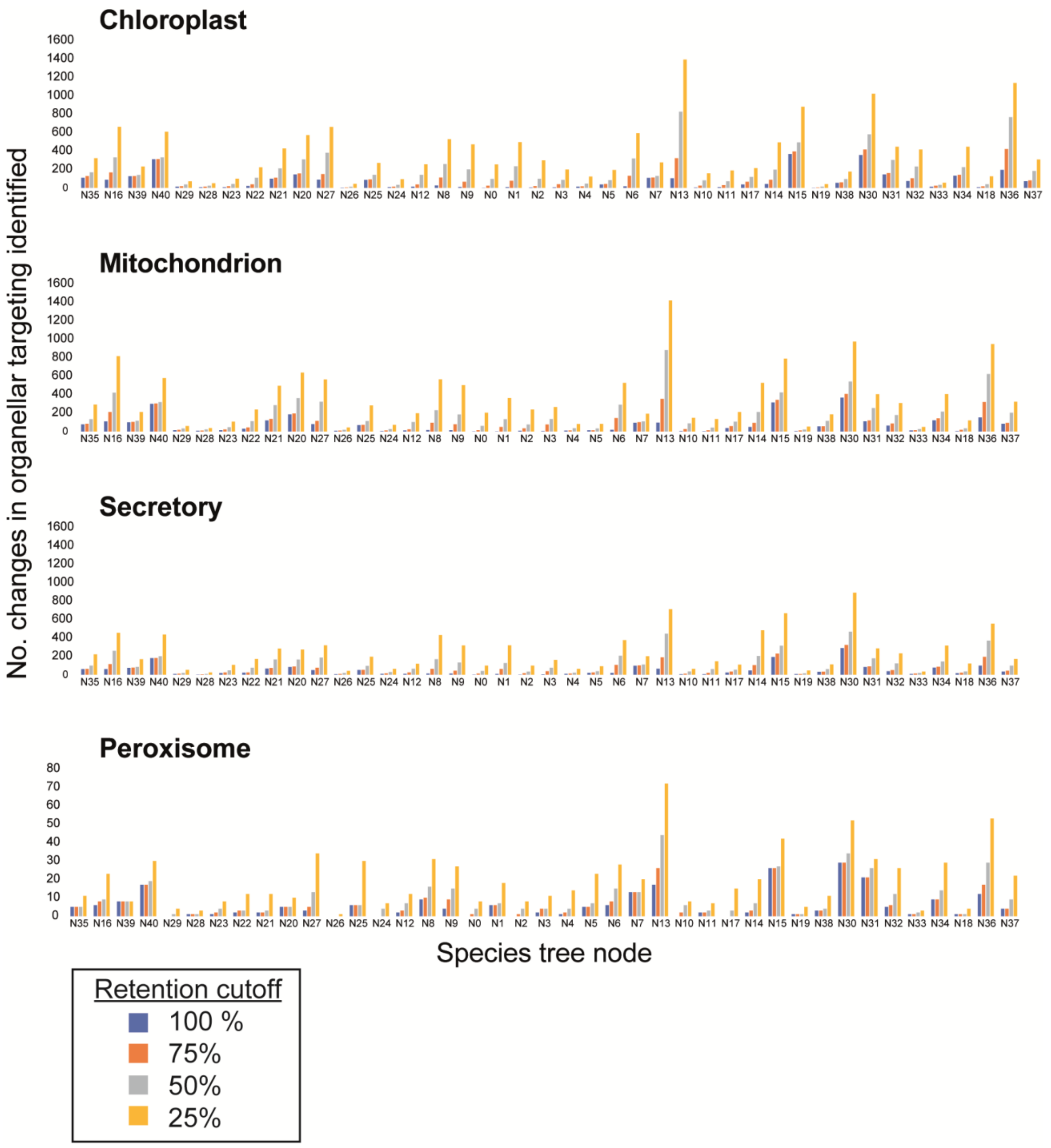

Figure S3. Recall of changes in organellar targeting using different retention scores for the proportion of genes which support a change. At its strictest, the retention score was 100%. Relaxation of the retention score to 75%, 50% and 25% resulted in more changes in organellar targeting

being identified each time. This is shown as the number of changes in organellar targeting identified for each of the species tree branches using the different retention cutoffs.

Figure S4

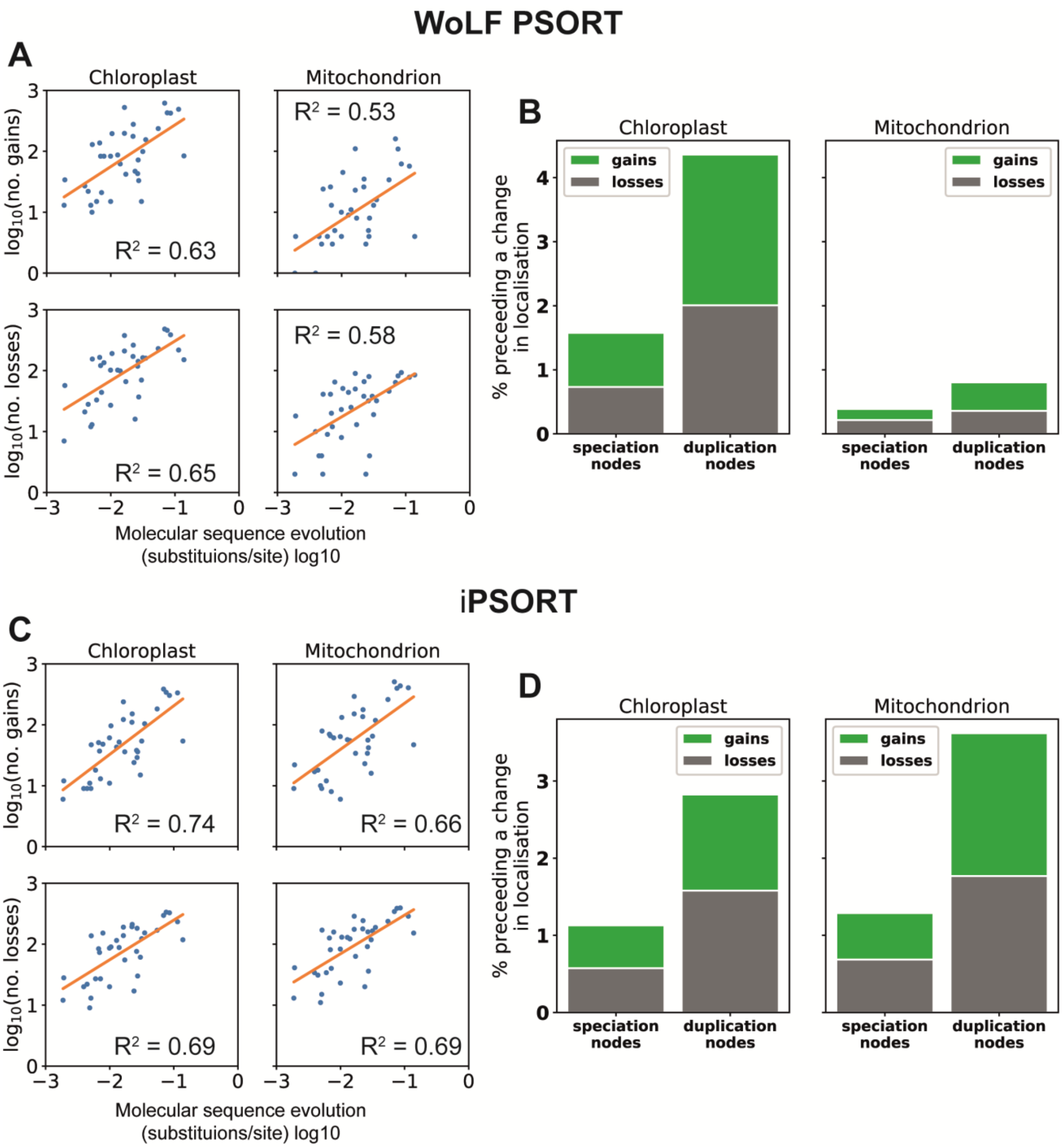

Figure S4. Comparison of protein prediction tools on the identification of changes in organellar targeting using ancestral character estimation. Wolfpsort (Horton et al. 2007) and IPSORT (Bannai et al. 2002) were used independently to predict chloroplast and mitochondrion proteins in orthgroups. **A** and **C:** there is a positive correlation between species tree branch length and the number of gains and

losses in protein targeting to the chloroplast and mitochondrion identified for that branch ($p<0.001$ for all plots). **B** and **D:** in both cases a higher proportion of orthogroup duplication nodes immediately proceed a gain or loss in organellar targeting compared to non-duplication (speciation) nodes ($p<0.001$, hypergeometric test).

Figure S5

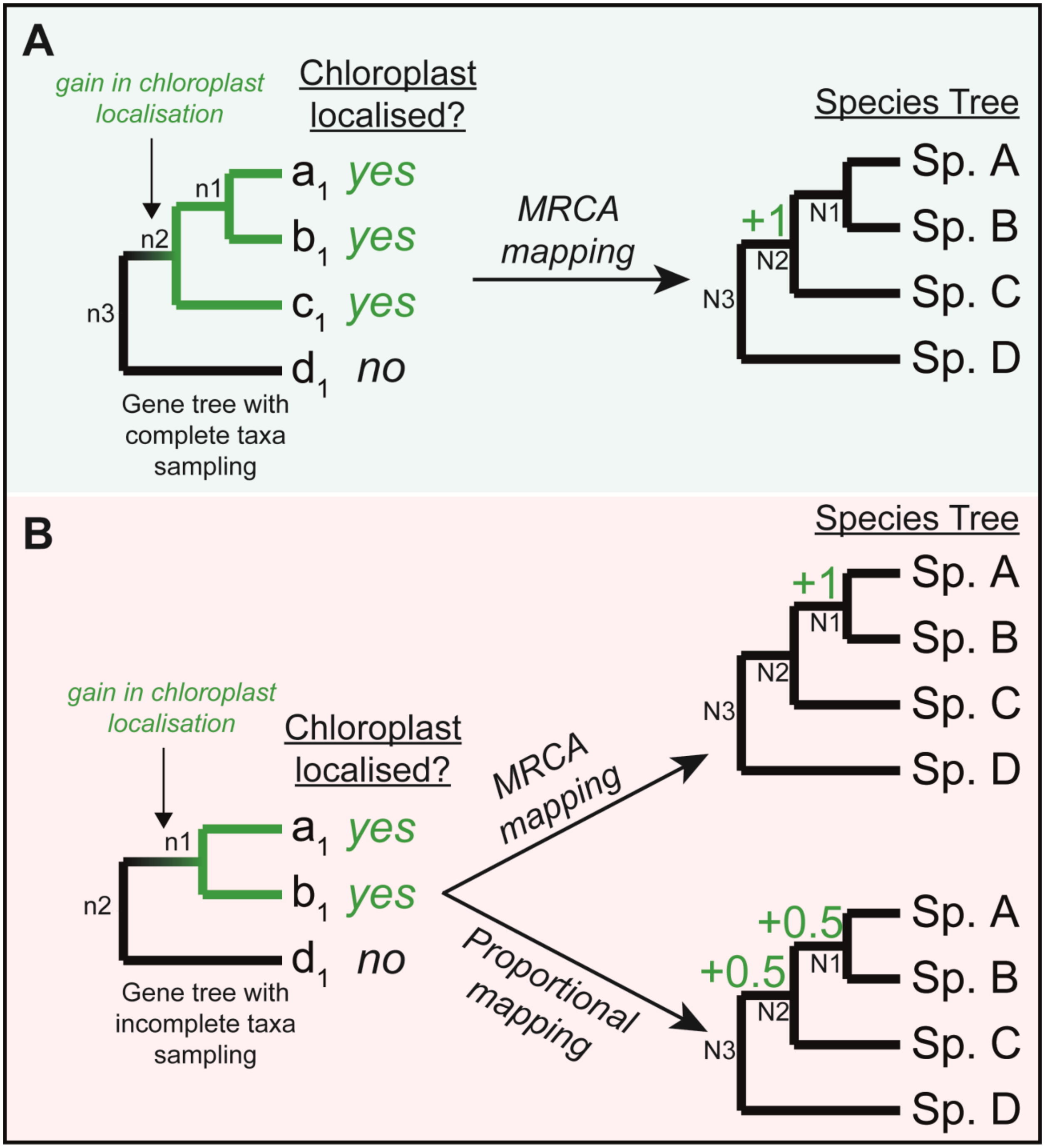

Figure S5. The effects of different gene-tree branch to species-tree branch mapping when genes are missing from a particular species. A: Gene-tree branches can be unambiguously mapped to the species using most recent common ancestor (MRCA) mapping when there is 100% representation of species within a gene clade (taxa sampling). In this example, ACE identifies a change in chloroplast targeting on n2 of the gene tree in the common ancestor of a1, a2 and a3. Given this, MRCA mapping

would add one (+1) to the tally of changes in chloroplast targeting in the ancestor of species A, B and C on the species tree (N2). **B:** When a gene is missing (either through true loss or missing gene models) such that there is no representation in a clade from one species, gene-tree branches cannot be mapped to just a single branch in the species tree. In this gene tree, the gene c1 is not present and so ACE now identifies a change in chloroplast targeting on n1 of the gene tree. If the gene c1 is missing due to a missing gene model in Species C, then without its predicted localisation it is impossible to know if the change occurred in the ancestor of a1 and b1, or in the ancestor of a1, b1 and c1. Using a MRCA approach would map the change in the ancestor of species A and B, adding +1 to the tally at N1. Alternatively, a proportional mapping approach can be taken where the change is mapped to both N1 and N2 of the species tree with the tally split evenly between these nodes, +0.5 to each.

Figure S6

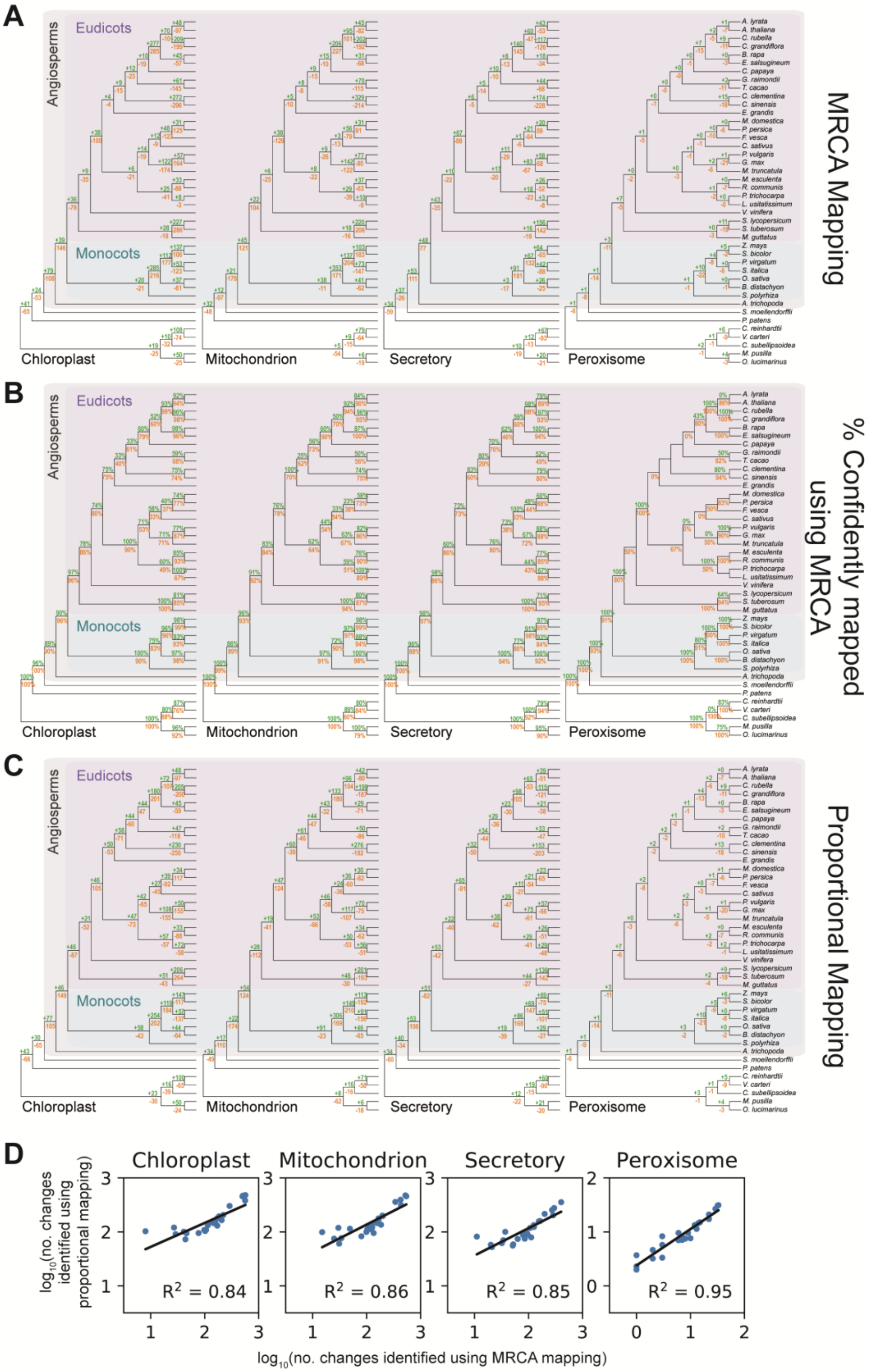
A
B
C
D
Eudicots
Monocots
Angiosperms
Chloroplast
Mitochondrion
Secretory
Peroxisome
MRCA Mapping
% Confidently mapped using MRCA
Proportional Mapping
A. lyrata
A. thaliana
C. rubella
C. grandiflora
B. rapa
E. salsugineum
C. papaya
G. raimondii
T. cacao
C. clementina
C. sinensis
E. grandis
M. domestica
P. persica
F. vesca
C. sativus
P. vulgaris
G. max
M. truncatula
M. esculenta
R. communis
P. trichocarpa
L. usitatissimum
V. vinifera
S. lycopersicum
S. tuberosum
M. guttatus
Z. mays
S. bicolor
P. virgatum
S. italica
O. sativa
B. distachyon
S. polyrhiza
A. trichopoda
S. moellendorffii
P. patens
C. reinhardtii
V. carteri
C. subellipsoidea
M. pusilla
O. lucimarinus
log10(no. changes identified using proportional mapping)
R² = 0.84
R² = 0.86
R² = 0.85
R² = 0.95
log10(no. changes identified using MRCA mapping)

Figure S6. Identification of uncertainty in the mapping of changes in organellar targeting to the species tree and comparison of MRCA and proportional mapping approaches. A: Changes in organellar targeting in orthgroup trees mapped to the species tree using a most recent common ancestor (MRCA) approach as shown in Fig. 2 of the main text. **B:** The percentage of changes mapped using the MRCA approach that can be confidently mapped to a single branch on the species tree. **C:** Changes in organellar targeting in orthogroup trees mapped to the species tree using a proportional mapping approach. **D:** The number of changes identified by both mapping approaches is strongly correlated across the species tree. R^2 values shown and $p<0.001$ for each correlation.

Figure S7

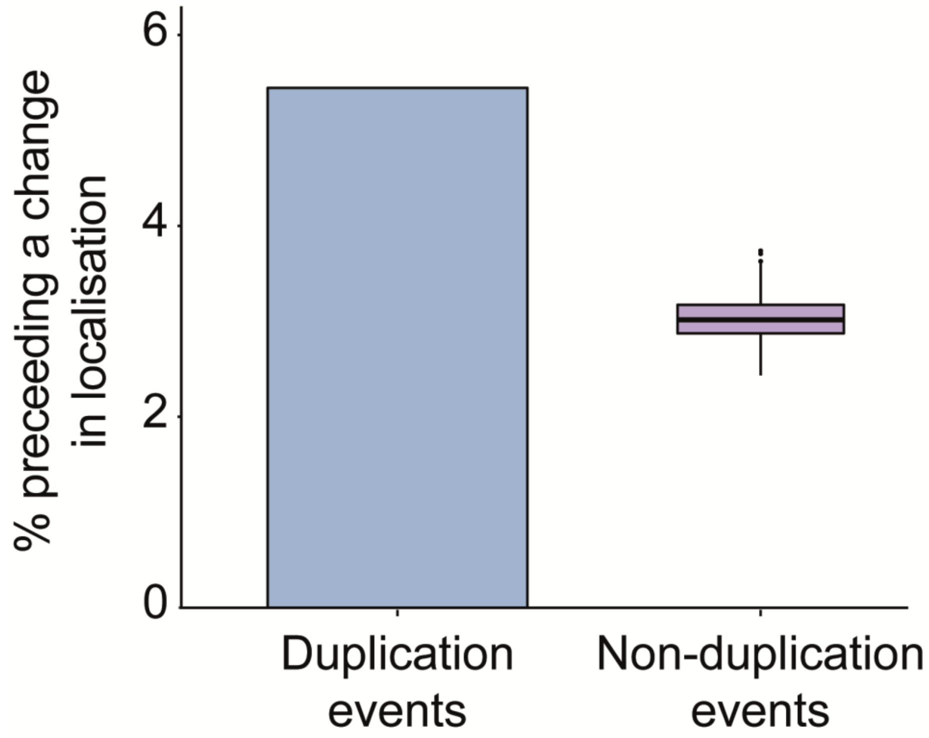

Figure S7. A higher proportion of gene duplication events are followed by a change in organellar targeting than non-duplication events. The percentage of genes that experienced a change in organellar targeting following gene duplication is shown by the bar on the left. These gene duplication events are distributed across the species tree. For each branch in the species tree, gene duplications were counted, and an identical number of speciation nodes mapped to the same branch were sampled at random. Thus, the proportion of duplication and non-duplication branches followed by a change in organellar targeting could be evaluated while controlling for phylogenetic placement of gene duplication events. This sampling was repeated 1000 times. The distribution of the proportion of non-duplication nodes followed by a targeting change from these 1000 samples is shown in the boxplot on the right (i.e. the box plot contains 1000 points). Given this distribution it can be determined that changes in protein targeting occur more frequently following gene duplication ($p<0.0001$). This supports the finding presented in Fig. 6A of the main text.

References

Bannai H, Tamada Y, Maruyama O, Nakai K, Miyano S. 2002. Extensive feature detection of N-terminal protein sorting signals. Bioinformatics.

Boussau B, Szollosi GJ, Duret L, Gouy M, Tannier E, Daubin V. 2013. Genome-scale coestimation of species and gene trees. Genome Res. [Internet] 23:323–330. Available from: http://www.ncbi.nlm.nih.gov/pubmed/23132911

Horton P, Park KJ, Obayashi T, Fujita N, Harada H, Adams-Collier CJ, Nakai K. 2007. WoLF PSORT: Protein localization predictor. Nucleic Acids Res.

Nguyen LT, Schmidt HA, Von Haeseler A, Minh BQ. 2015. IQ-TREE: A fast and effective stochastic algorithm for estimating maximum-likelihood phylogenies. Mol. Biol. Evol.

Salichos L, Rokas A. 2013. Inferring ancient divergences requires genes with strong phylogenetic signals. Nature.

Schwarte S, Bauwe H. 2007. Identification of the photorespiratory 2-phosphoglycolate phosphatase, PGLP1, in Arabidopsis. Plant Physiol. [Internet] 144:1580–1586. Available from: https://www.ncbi.nlm.nih.gov/pmc/articles/PMC1914141/pdf/pp1441580.pdf

Swenson KM, Doroftei A, El-Mabrouk N. 2012. Gene tree correction for reconciliation and species tree inference. Algorithms Mol. Biol.

Szllosi GJ, Tannier E, Daubin V, Boussau B. 2015. The inference of gene trees with species trees. Syst. Biol.

Tardif M, Atteia A, Specht M, Cogne G, Rolland N, Brugière S, Hippler M, Ferro M, Bruley C, Peltier G, et al. 2012. Predalgo: A new subcellular localization prediction tool dedicated to green algae. In: Molecular Biology and Evolution. Vol. 29. p. 3625–3639.
